# P238: Surveillance in geriatrics: what for? Which indicators?

**DOI:** 10.1186/2047-2994-2-S1-P238

**Published:** 2013-06-20

**Authors:** V Sauvan, Y Registe Rameau, L Pagani, D Pittet, S Harbarth

**Affiliations:** 1 Infection Control Program, Geneva University Hospitals, Geneva, Switzerland; 2 DMIRG, Geneva University Hospitals, Geneva, Switzerland

## Objectives

Surveillance of epidemiologically important pathogens and measurement of related indicators are key tasks of Infection Control staff (ICS). Active surveillance is carried out by ICS in a 300-bed geriatric hospital in Switzerland to improve safety of hospitalized elderly patients.

## Methods

The following surveillance activities are currently carried out: monitoring of patients colonized with MRSA, including sampling and collection of new, non-endemic strains of MRSA, notably carrier of SCCmecII between 3/2011 and 12/2012; epidemiology and investigation of any new proven case of *C. difficile* infection; investigation of viral infection outbreaks (norovirus since 2008, and influenza since 2011). Compliance with hand hygiene practices is monitored since 2006. In addition, in 2012, we performed an evaluation on indicators of: correct disinfection of multiple-usage medical devices; correct application of specific isolation measures; environmental cleansing along the course of viral gastroenteritis clusters or *C. difficile* infection.

## Results

MRSA incidence rate (n. of new cases/100 admissions) remained around 3% in 2012. Introduction and cross-transmission of new MRSA strains carrying SCCmecII has been identified. Between 18/12/12 and 02/01/13, an outbreak of norovirus gastroenteritis occurred, with 130 nosocomial cases and 33 cases among healthcare workers. Fifty-two nosocomial cases out of 85 total cases of influenza infection were recorded in the winter season 2012-13. Incidence of *C. difficile* infection remains high, with 15 new cases/1000 admissions in 2012. Overall adherence to hand hygiene remains around 70%. Compliance with disinfection of non-disposable medical devices is 100% after the implementation of an educational program. Yearly audit of application of isolation measures shows now a correct patient placement in around 90% of cases, whereas audit on quality of environmental cleansing has shown insufficient adherence.

## Conclusion

Infection control and prevention remain challenging within a geriatric hospital, mostly for viral infections and *C. difficile*. Both active surveillance and indicators reporting help improve healthcare quality and patient safety in this setting.

## Disclosure of interest

None declared

